# Cellular senescence and other age-related mechanisms in skeletal diseases

**DOI:** 10.1038/s41413-025-00448-7

**Published:** 2025-07-07

**Authors:** Ke Li, Sihan Hu, Hao Chen

**Affiliations:** 1 Institute of Translational Medicine, Medical College, Yangzhou University, Yangzhou, PR China; 2 The Key Laboratory of the Jiangsu Higher Education Institutions for Nucleic Acid & Cell Fate Regulation, Yangzhou, PR China

**Keywords:** Pathogenesis, Bone

## Abstract

Cellular senescence and its senescence-associated secretory phenotype (SASP) represent a pivotal role in the development of skeletal diseases. Targeted elimination or rejuvenation of senescent cells has shown potential as a therapeutic strategy to reverse age-related skeletal senescence and promote bone regeneration. Meanwhile, other age-related mechanisms, involving altered cellular functions, impaired intercellular crosstalk, disturbed tissue microenvironment, and decreased regenerative capacity, synergistically contribute to the pathogenesis. In this review, we outline the cellular senescence and other age-related mechanisms in developing skeletal diseases, including osteoporosis, intervertebral disc degeneration, osteoarthritis, rheumatoid arthritis, bone tumors and ankylosing spondylitis, with the aim of comprehensively understanding their detrimental effects on the aged skeleton and screening the potential targets for anti-aging therapy within the skeletal system.

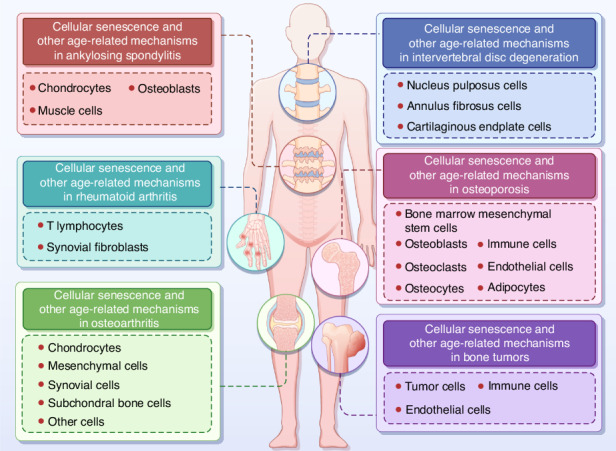

## Introduction

The skeleton, as a metabolically active and continuously remodeling tissue, serves to protect internal organs and support body exercise.^1^ It also has active endocrine functions that closely regulate the health of the body. As a reservoir of various types of cells, the collaborative efforts of different cells in the skeletal system promote the establishment of bone homeostasis.^2^ However, cellular senescence and other age-related mechanisms synergistically lead to impaired bone cell function, facilitating the onset and progression of bone diseases, such as osteoporosis (OP), intervertebral disc degeneration (IVDD), and osteoarthritis (OA). Cellular senescence is a particular cellular state characterized by irreversible arrest of the cell cycle and the emergence of a distinctive senescence-associated secretory phenotype (SASP), which is one of the key mechanisms in the development and progression of skeletal diseases.^3^ In addition, other age-related mechanisms occur in the skeletal system that synergistically contribute to the development of bone diseases. For example, the impaired intercellular crosstalk leads to an abnormal accumulation of senescence phenotypes in the bone marrow,^4^ the generation of a disturbed microenvironment promotes senescence in the skeletal system.^5^

**Fig. 1:**
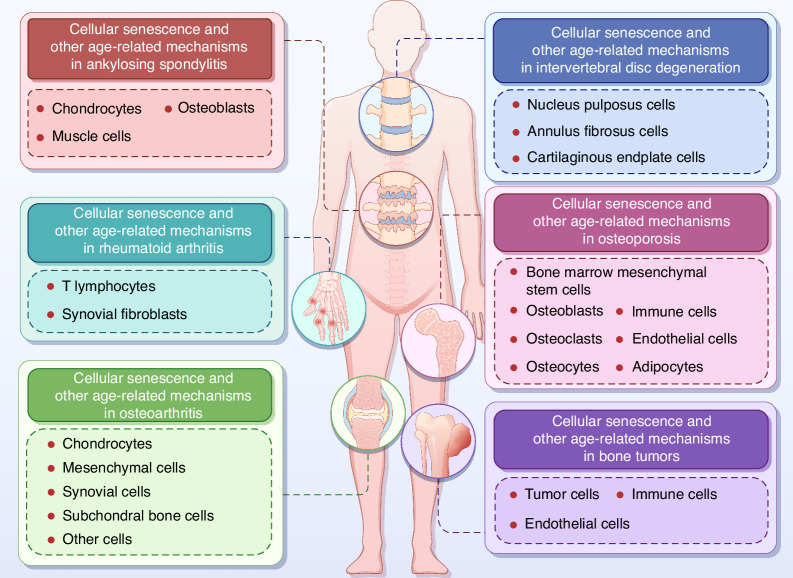
Cellular senescence and other age-related mechanisms in skeletal diseases. The synergistic action of various cells maintains bone homeostasis. Cellular senescence and other age-related mechanisms lead to impaired function of bone cells, contributing to the onset and progression of bone disorders, including osteoporosis (OP), intervertebral disc degeneration (IVDD), osteoarthritis (OA), bone tumors, and ankylosing spondylitis (AS)

The elderly population has a high prevalence of age-related bone diseases.^6^ Particularly in elderly individuals (aged 65 and above), age-related bone diseases represent the leading cause of disability worldwide.^7^ The associated pain and limited mobility lead to a decrease in quality of life, posing a significant healthcare burden to the government.^8^ Prospects for the development of drugs targeting skeletal aging diseases are currently not optimistic, largely owing to a lack of understanding of the cellular senescence and other age-related mechanisms that lead to bone dysfunction during aging.^9^ In this review, we summarize the current understanding of cellular senescence and other age-related mechanisms in the pathogenesis of bone diseases, highlighting the diversity of the mechanisms involved in cellular senescence within different skeletal aging microenvironments. Moreover, we provide an overview of therapeutic approaches involving selective elimination or the reversion of cellular senescence (Fig. 1).

## Cellular senescence and other age-related mechanisms in osteoporosis

As a complex and dynamic organ, bone is maintained by the coordination of multiple cell types, including bone marrow mesenchymal stem cells (BMSCs), osteoblasts (OBs), osteoclasts (OCs), osteocytes (OCYs), adipocytes (ADs), immune cells, etc.^10^ As rich cellular reservoirs, the various bone cells in the bone marrow undergo a cellular change and cause bone aging as the lifespan increases, ultimately leading to the loss of bone integrity and the development of OP.^11,12^ The cellular senescence and other age-related mechanisms cause OP through various mechanisms involving different cell types.

### Bone marrow mesenchymal stem cells

Recruiting BMSCs to sites of bone destruction and differentiating them toward osteogenic lineage is an approach for maintaining bone homeostasis.^13^ BMSCs obtained from elderly individuals exhibit replicative senescence and decreased osteogenic potential.^14^ Advancing age induces changes in the differentiation potential of BMSCs, with a tendency toward adipogenic differentiation, leading to a lack of new bone supply and the accumulation of ADs in the bone marrow.^15^

Single-cell RNA sequencing (scRNA-seq) analysis has recently been applied to reveal changes in the characteristics of BMSCs during aging. BMSCs labeled with fibroblast growth factor receptor 3 (Fgfr3)-creER exhibit strong colony-forming ability at a young age, but this ability is lost when these cells are aged.^16^ In addition, leptin receptor (LepR)-cre-labeled BMSCs presented an increase in the proportion of colony-forming cells with advancing age.^16^ These changes may reduce the osteogenic differentiation of BMSCs during aging and contribute to the development of OP. Senescence triggers the differentiation of C-X-C motif chemokine ligand 12^+^ (CXCL12^+^) LepR^+^ BMSCs toward a lipogenic cell lineage, which provides a rationale for ADs accumulation in aged bone marrow.^17,18^ Recent work has provided an in-depth analysis of human BMSCs (hBMSCs) via scRNA-seq, which classified these cells into 10 distinct subpopulations. In particular, functional enrichment analysis of a specific sub-population expressing P62 revealed a series of biological processes, including cellular redox homeostasis, the endoplasmic reticulum stress response, and the inhibitory regulation of cell growth, that may be involved in BMSC aging.^19^ scRNA-seq also revealed a notable decline in the paired related homeobox 1^Cre+^Osterix^Cre−^ (Prx1^Cre+^Osx^Cre−^) sub-population of BMSCs in Prx1-Cre; Zmpste24^f/f^ (Z24^f/f^) mice that exhibited premature aging.^20^ This sub-population has been observed to undergo activation of apoptotic signaling pathways and a reduction in mechanosensation, both of which are strongly linked to age-related bone loss. hBMSCs with premature aging syndrome were generated by knocking out Werner syndrome protein, and they exhibited reduced self-renewal capacity and increased senescence-associated-β-galactosidase (SA-β-gal) activity. A genome-wide CRISPR activation screen utilizing hBMSCs in a senescence model demonstrated that the deletion of sry-box transcription factor 5 (SOX5) resulted in a reduction in the enhancer activity of high mobility group box 2 (HMGB2) and a decrease in the levels of H3K27ac and H3K4me1, thereby facilitating cellular senescence.^21^ Data support the hypothesis that the in vitro and in vivo inhibition of transcription factor E Box-binding (TFEB) nuclear translocation-mediated autophagy results in the induction of a senescent state in BMSCs, thereby contributing to the development of aging-associated bone loss.^22^ Recent studies have proposed that epigenetic changes are also significant causes of aging.^23–25^ The expression of the DNA methyltransferases DMNT-1 and DNMT-3B, which increase the expression of p16 and p21, is significantly reduced in senescent BMSCs. In senescent BMSCs, a significant increase in the active chromatin marker H3K4me1 is found in hypomethylated regions, which underscores the role of histone methylation in the process of age-related cellular senescence.^26^

Extracellular vesicles (EVs) secreted by skeletal myocytes are taken up by BMSCs through the blood circulation. A significant increase in the expression of miRNA-34a-5p has been observed in EVs derived from both senescent human myotubular cells and mouse skeletal muscle cells, which induces senescence in BMSCs.^4^ A study further demonstrated that lactate dehydrogenase in EVs secreted by young skeletal muscle cells promotes osteogenesis by activating the glycolytic pathway in BMSCs,^27^ thus establishing the foundation for bone loss therapies for the elderly based on skeletal muscle cell-BMSCs crosstalk. Endothelial cells (ECs), immune cells, and BMSCs are closely related to the OP microenvironment. Vascular ecological niche analyses in older populations and mice demonstrated increased secretion of miRNA-31-5p in the senescent ECs, which inhibits BMSCs’ osteogenesis by suppressing wingless-related integration site (WNT) signaling.^28^ Macrophages (MACs) in the OP microenvironment release oxidatively damaged mitochondria. The transfer of mitochondria from MACs to BMSCs triggers a burst of reactive oxygen species (ROS), leading to restricted osteogenic differentiation of BMSCs.^29^ Notably, senescent BMSCs show increased SASP secretion, which promotes the aged bone marrow microenvironment.^30^ Senescent BMSCs also facilitate OCs maturation and myeloid skewing of hematopoietic stem and progenitor cells via the SASP.^5^ These findings provide important insights into the functional changes in BMSCs during aging and may provide new targets and approaches to promote bone regeneration.

### Osteoblasts

OBs are intermediate cells derived from BMSCs’ osteogenic differentiation, and their senescence is closely associated with abnormal bone metabolism and OP occurrence.^31,32^ Senescent OBs may help form a malignant microenvironment, as young mice injected with OBs from aged mice exhibit severe bone loss.^33,34^ Notably, after OBs from young mice were directly injected into the bone marrow cavity of aged mice through bone marrow transplantation, the aged mice presented increased histological trabecular bone and bone mineral density.^35^ The reversal of OB senescence has potential in the treatment of OP.

One recent study revealed that CD24^+^ OBs exhibit significant growth arrest, SA-β-gal positivity, and osteogenesis restriction, as shown by time-of-flight mass cytometry.^36^ Investigators have demonstrated that the elimination of CD24^+^ OBs in the aged mice suggests the concept that senescent cell elimination may improve aspects of skeletal aging. The production of lymphoid progenitor cells in the periphery was found to be supported by a specific type of osteoblastic progenitor cell in the bone marrow, namely osteolectin-positive (Osteolectin^+^) cells. Following reduced exercise due to aging, Osteolectin^+^ cells exhibit less proliferation, decreased bone formation, and reduced lymphocytes, which ultimately lead to poor bone quality and diminished immune function.^37^ This population of cells provides a novel therapeutic target for intervention in bone aging. The senescence of OBs is regulated by multiple pathways. Mechanistic target of rapamycin complex 1 (mTORC1) was demonstrated to induce membrane potential depolarization by modulating the sodium channel Scn1a, thereby accelerating pre-OBs senescence by increasing Ca²^+^ influx and activating downstream NFAT/ATF3/p53 signaling.^38^ It has been shown that methyltransferase-like 3 (METTL3) deficiency in senile OP promotes OB senescence by decreasing Hspa1a mRNA stability in a YTH N6-methyladenosine RNA-binding protein 2 (YTHDF2)-dependent manner.^39^ In the past decade, the “oxidative stress theory of aging” has attracted widespread attention. The excessive accumulation of ROS in the bone marrow microenvironment induces OB malfunction by activating the forkhead box O (FOXO) pathway and blocking Wnt signal transduction.^40,41^ Moreover, senescent OBs exhibit reduced responsiveness to insulin-like growth factor (IGF)-I, thereby inhibiting osteogenic proliferation and differentiation.^42^

Direct cell-to-cell contact between OBs and OCs is achieved through the interaction of the Ephrin B2 (EFNB2)-EPHB4, FAS-FAS ligand (FASL), and Neuropilin-1 (NRP1)-Semaphorin 3A (SEMA3A) signaling pathways, which in turn regulate cell proliferation and differentiation.^43^ In the bone microenvironment, OBs secrete a variety of key factors, including macrophage colony-stimulating factor (M-CSF), receptor activator of nuclear factor-κB ligand (RANKL), and WNT5A, which promote OCs formation.^43^ An RNA sequencing data suggests that schnurri-3 (SHN3) secreted by OBs inhibits H-type blood vessel formation through negative regulation of the transcription and expression of the angiogenic factor SLIT3, consequently contributing to the development of OP.^44^ The present work reveals the OBs-ECs crosstalk and its important role in bone regeneration. Substantial evidence suggests that the OBs-immune cell crosstalk also plays a central role in osteogenesis. The CD39-CD93-adenosine receptor (AdoR) signaling pathway, which is reduced by Treg cells, directly inhibits OBs’ osteogenesis.^45^ Future studies need to further explore the potential cellular and molecular mechanisms linking OBs senescence and OP development, hopefully providing new insights into the development of targeted therapeutic strategies and new perspectives for clinical treatment.

### Osteoclasts

Aged bone shows enhanced bone resorption mediated by hematopoietic-derived OCs under osteoporotic conditions. In aged animals, OCs are overactivated, resulting in a greater capacity for bone removal than young bones do, which ultimately leads to negative bone homeostasis in the aged skeletal system.^46–48^ The development of OCs is dependent on crosstalk with OBs, OCYs, BMSCs, and even ADs. Reports indicate that increased secretion of RANKL and M-CSF, as well as decreased secretion of osteoprotegerin (OPG), in OBs, OCYs, and BMSCs in aging individuals promote OCs maturation and activation.^49–52^ Therefore, it seems that aging promotes increased OCs abundance and activation by regulating the RANKL/OPG axis, which leads to accelerated bone loss. In addition, bone marrow ADs have been shown to act on OCs activation through RANKL secretion.^53,54^ Unfortunately, the mechanism of ADs-OCs crosstalk remains to be elucidated in elderly individuals, and its role in bone aging requires further investigation.

Recent findings have confirmed a novel form of communication between OCs and BMSCs. Analysis of OCs-derived EVs has revealed an enrichment of thrombin cleavage-secreted phosphoprotein 1 (SPP1), and SPP1 deficiency inhibits osteogenic differentiation of BMSCs by reducing TGFβ1/SMAD3 signaling.^55^ Furthermore, OCs have been shown to influence OBs’ behavior by secreting different soluble factors. On the one hand, RANKL-stimulated OCs secrete more sphingosine 1 phosphate (S1P), which binds to the S1P receptor on the surface of OBs, leading to increased OBs proliferation and bone formation.^56^ Collagen triple helix repeat containing 1 (CTHRC1) released by OCs also induced OBs differentiation by targeting stromal cells, and knockdown of CTHRC1 expression in OBs led to a significant decrease in bone formation.^57^ On the other hand, OCs-derived semaphorin 4D (SEMA4D) binds to plexin-b1 (PLXNB1) on the surface of OBs, inhibiting OBs differentiation and accelerating bone loss.^58^ Studies have identified angiopoietin (ANG) secreted by OCs as a key factor in preventing senescence of neighboring ECs.^59^ ANG inhibits ECs’ senescence through plexin B2 (PLXNB2)-mediated ribosomal RNA transcription. OCs-ECs crosstalk provides a new molecular basis for studies of bone and vascular regeneration. Notably, changes in the bone matrix also contribute to changes in OCs activity. The presence of type I collagen β-isomerization of C-telopeptide in the bone matrix is considered to induce OC activation, and its content is three times greater in aged bones than in young bones.^60^ These findings suggest that changes in the aged bone matrix enhance the ability of OCs to resorb. Interestingly, in vitro experiments did not reveal an increase in or activation of OCs when these cells were co-cultured with the aged bone matrix, possibly because of the lack of a bone marrow microenvironment.^61^ OCs also promote OBs-mediated bone formation through the secretion of transforming growth factor (TGF)-β and IGF-1 secreted in the bone matrix.^43^ Crosstalk between OCs and other cells contributes to the regulation of OC activity and thus affects bone resorption. Hence, manipulating the crosstalk between various cells and OCs in the aging bone marrow is expected to be a potential therapeutic target for future treatment of OP.

### Osteocytes

OCYs account for more than 90% of the cells in the skeletal system, and their dendritic structures provide the basis for communication with other cells.^62^ OCYs maintain bone homeostasis by responding to mechanical loads and hormonal stimuli in the bone marrow.^63^ Research has demonstrated that reduced activity and decreased mechanical loading in elderly individuals lead to severe decreases in bone density.^64^ Numerous evidence have demonstrated that senescent OCYs accelerate bone loss by secreting SASP factors.^65,66^ Farr et al. identified more than 20 SASP genes that are significantly expressed in senescent OCYs from both humans (aged 72–87 years) and mice (aged 24 months).^11^ Additionally, the connection between autophagy and OCYs senescence has been elucidated. Atg7 and Map1lc3a, which are highly expressed mitochondrial autophagy genes in OCYs, are expressed at decreased levels in aged animals.^11^ Reduced mitochondrial autophagy promotes the accumulation of damaged mitochondria and ROS in OCYs, further contributing to OCYs’ senescence.^67^

Compared with healthy OCYs, senescent OCYs exhibit impaired mechanical signaling and disruption of perilacunar/canalicular remodeling (PLR).^10^ OCYs actively remodel their surroundings by reabsorbing and redepositing the surrounding bone matrix via PLR.^68,69^ The presence of PLR is confirmed by “OCYs osteolysis”, which manifests as enlarged lumina and rough borders around OCYs.^70^ Increasing evidence of reduced luminal volume and hypermineralized lacunae in older human and mouse bones strongly suggests that PLR is impaired in senescent OCYs.^63,71^ Furthermore, RNA-seq data from OCYs-rich cortical bone indicate that the expression levels of bone resorption-related genes cathepsin K (Ctsk) and tartrate-resistant acid phosphatase (Acp5) are significantly lower in senescent OCYs than in young OCYs.^72^ These findings suggest that OCs markers in OCYs are progressively lost during aging, which leads to the impaired PLR function of OCYs and the inhibited activation of OCs. In addition, senescent OCYs also activate OCs by secreting M-CSF and RANKL and inhibit the function of OBs by secreting sclerostin.^73^ Young OCYs-derived EVs and senescent OCYs-derived EVs (S-EVs) were obtained from primary OCYs of 2-month-old and 16-month-old mice, respectively. Proteomic analysis showed that S-EVs lack promyosin-1 (TPM1), which promotes osteogenic differentiation of BMSCs.^74^ In-depth exploration of aging-induced cellular senescence in OCYs provides potential strategies for the treatment of senile OP.

### Adipocytes

Substantial research confirms the regulatory role of bone marrow ADs in skeletal health, as their accumulation leads to bone loss.^75^ This phenomenon may be attributed to changes in the fate of BMSCs differentiation during aging. Although age-dependent accumulation of ADs in the bone marrow resulting in OP has been observed in elderly individuals, exploration of signaling pathways is still in the early stage.^76^

Studies have revealed that dipeptidyl peptidase-4 (DPP4) derived from ADs in aged bone marrow has a significant osteogenic inhibitory effect. Conversely, a significant increase in bone mass was observed upon targeted removal of DPP4.^17^ Moreover, bone marrow ADs produce more receptor activators for RANKL in response to parathyroid hormone deficiency or glucocorticoid intervention, leading to abnormal activation of OCs and bone resorption.^77,78^ Notably, a recent study explored the mechanisms of senescent ADs in the bone marrow and revealed that bone marrow ADs transform into senescent ADs after glucocorticoid administration. The secretion of SASP from senescent ADs further promotes secondary senescence of ECs and OBs in the skeleton. Mechanistically, the formation of a positive feedback loop of 15d-PGJ2-PPARγ-INK signaling in the skeletal system ultimately leads to bone degeneration.^75^ In addition, the expression level of bone marrow macrophage-derived proliferating cell nuclear antigen clamp-associated factor (PCLAF) was found to be significantly elevated in aged mice compared with young mice. PCLAF binds to the ADGRL2 receptor on bone marrow ADs, promoting bone marrow AD senescence and disrupting bone homeostasis.^79^ These studies have sparked strong interest in senescent ADs in the development of OP and indicate that clearing mediators derived from senescent ADs may be an effective approach to alleviate OP.

### Immune cells

In addition to the negative impact of senescent bone cells in the skeleton, immune cells in elderly individuals also participate in the regulation of bone homeostasis. Elderly individuals exhibit chronic inflammation and changes in the immune cell spectrum.^80^ Previous studies have revealed that various immune cells establish communication with bone cells through direct contact or paracrine pathways.

MACs and neutrophils (NEs) accumulate and secrete large amounts of grancalcin (GCA) protein in the bone marrow during aging, and GCA binds to Plexin-B2, a cell surface receptor for BMSCs in the bone marrow, and inhibits the downstream FAK-SRC-YAP signaling pathway, which inhibits the osteogenic differentiation of BMSCs.^81^ Injection of GCA antibody into aged mice resulted in an increase in the number of OBs and OCs on the surface of the bone trabeculae and a significant decrease in the number and area of ADs. These findings suggest that the GCA antibody significantly improves the skeletal health of aged mice. Furthermore, inflammatory factors from M1 MACs and anti-inflammatory factors secreted by M2 MACs play important roles in the fate of bone cells.^82^ Non-polarized MACs can also promote the differentiation of BMSCs into the OBs lineage via oncostatin M (OSM).^83^ TGFβ1^+^CCR5^+^ NEs were reported to be the major source of TGFβ1 in mouse bone marrow.^84^ With age, increased TGFβ1^+^CCR5^+^ NEs in the bone marrow induce TNF receptor-associated factor 3 (TRAF3) degradation in mesenchymal progenitor cells via TGFβ1, which promotes NF-κB-mediated CCL5 expression, ultimately leading to bone loss. NEs infiltrating the aging bone marrow also induce OCs formation through the secretion of RANKL and IL-8.^85^ T lymphocytes are closely related to T lymphocyte subsets and cytokines in regulating bone regeneration. T help 1 (Th1) cells obtained from aged animal models show increased production of tumor necrosis factor-α (TNF-α), which promotes OBs apoptosis and mediates B cell secretion of RANKL, leading to OCs activation and severe bone loss.^86^ Interleukin 17 (IL-17) secreted by Th17 cells upregulates RANKL expression on the surface of pre-OCs, BMSCs, and OBs, thereby promoting OCs maturation and inhibiting OBs differentiation.^87^ T regulatory (Treg) cells promote osteogenic differentiation of pre-OBs and BMSCs directly through the CD39-CD93-AdoR signaling pathway.^45^ Additionally, Treg cells inhibit OCs’ function by secreting cytotoxic T-lymphocyte-associated protein 4 (CTLA4) and activating the CD80/CD86 receptors on pre-OCs.^88^ These data indicate that T lymphocytes and their derived mediators constantly influence bone balance. Clinical data also revealed that B lymphocytes in the bone marrow of elderly individuals are either normal or reduced, accompanied by increased secretion of G-CSF and RANKL.^89,90^ This is inextricably linked to the increased formation and activation of OCs. In summary, the crosstalk between immune cells and bone cells directly or indirectly influences the fate of bone cells. Exploring the molecular mechanisms between immune cells and bone cells is beneficial for alleviating the progression of OP.

### Endothelial cells

Evidence suggests that angiogenesis and osteogenesis are commonly coupled in the mammalian skeletal system. However, reduced angiogenesis and osteogenesis are observed in aged organisms and ultimately lead to the development of OP.^59,91,92^ ECs are highly heterogeneous in different tissues and acquire specific morphological, molecular, and functional properties in the local microenvironment.^93,94^

In recent years, researchers have reported that in mouse bones, capillaries can be differentiated into H-type (CD31^high^/Emcn^high^) and L-type (CD31^low^/Emcn^low^) ECs, which are categorized on the basis of their shape, molecular properties, and function.^91^ In particular, H-type ECs, located at the end of the skeletal arterial network, may play a central role in the specialized metabolic organization of the environment. H-type ECs enjoy preferential access to oxygen and nutrients, which may have an important impact on the senescence and regeneration of other cell types. Studies have also shown that H-type ECs provide essential signals to surrounding OBs through the HIF and Notch signaling pathways. H-type ECs are expressed at significantly lower levels in bone with age, which may provide a plausible explanation for OP.^91^ Moreover, the inhibition of epiphyseal ECs’ senescence ameliorates impaired bone angiogenesis and the decoupling of the osteogenic process through the ANG/PLXNB2 signaling axis.^59^ Decreased production of pre-OCs in the epiphysis results in a significant decrease in ANG secretion, which leads to impaired rRNA transcription in ECs, ultimately causing aging-related cellular senescence. Recent studies have demonstrated that senescent ECs-derived miRNA-31-5p promotes BMSC lipogenic differentiation by regulating the WNT pathway.^28^ Conversely, young ECs-exo upregulate zinc finger and BTB domain containing 16 (ZBTB16) expression to promote BMSCs osteogenic differentiation.^95^ These findings confirmed that senescent ECs and bone cells mediate angiogenesis and osteogenesis in the aged skeleton.

**Fig. 2:**
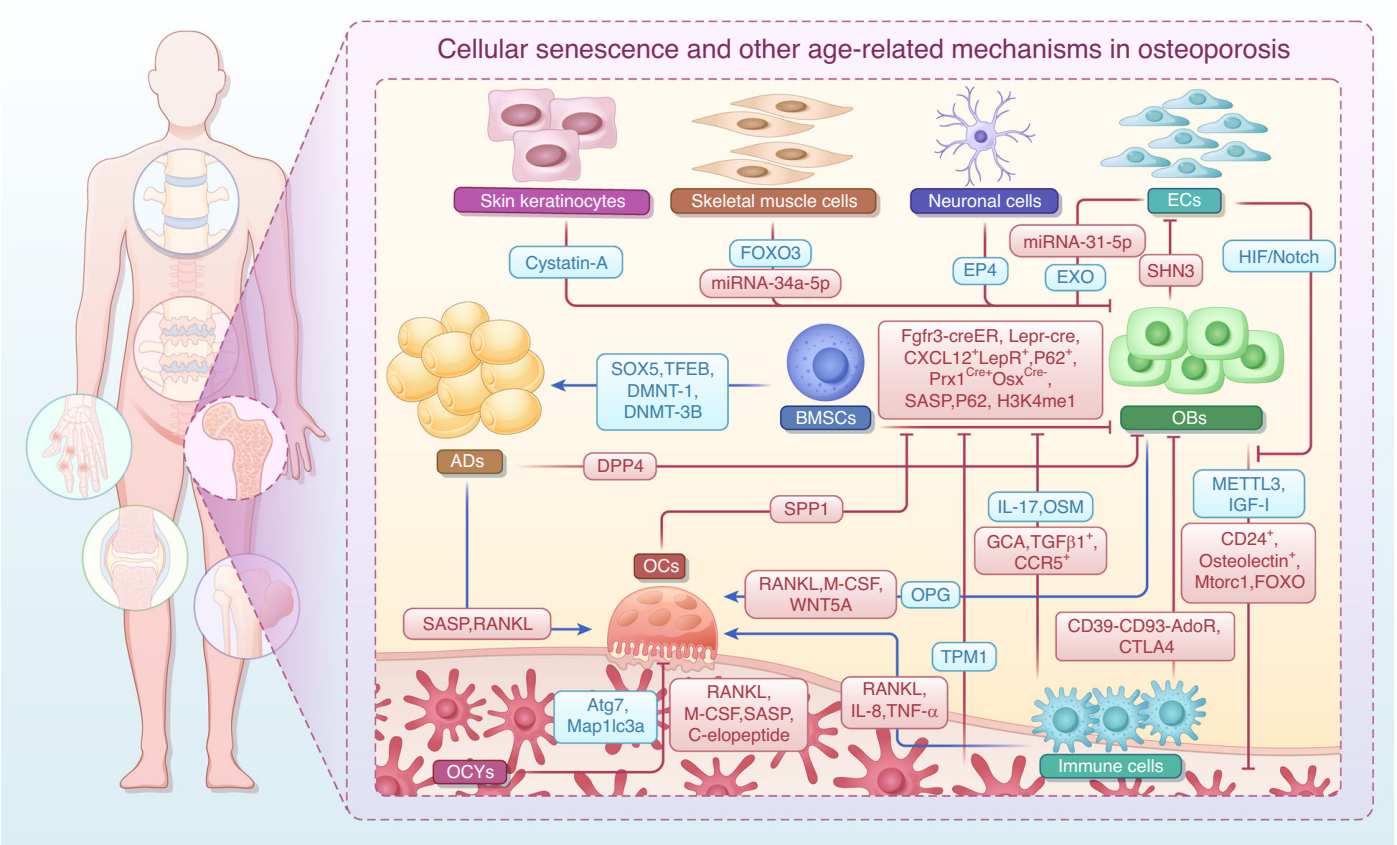
Cellular senescence and other age-related mechanisms in the pathogenesis of OP. The senescence of different bone cells and OP is interwoven into a complex network of interactions. Senescent cells co-secreted the SASP to accelerate inflammatory microenvironment formation and bone loss. Fgfr3-creER, Lepr-cre, CXCL12^+^LepR^+^, P62^+^, and Prx1^Cre+^Osx^Cre−^ labeled BMSCs were shown to be involved in the biological process of BMSCs senescence. Moreover, senescent BMSCs also showed increased expression of P62 and H3K4me1 and decreased expression of SOX5, TFEB, DMNT-1, and DNMT-3B. In addition, the senescence of BMSCs is induced by miRNA-34a-5p secreted by senescent skeletal muscle cells and by senescent ECs through the secretion of miRNA-31-5p. The OBs were differentiated osteoblastically from BMSCs. CD24^+^ and Osteolectin^+^ OBs exhibited a distinct senescent phenotype and osteogenic restriction. Increased mTORC1 and FOXO and decreased METTL3 and IGF-I in senescent OBs induced malfunction in OBs. Senescent OBs promoted OCs activation through massive secretion of M-CSF, RANKL, and WNT5A. SHN3 secreted by OBs promoted OP development through the inhibition of H-type angiogenesis. Treg cell-mediated inhibition of the CD39-CD93-AdoR signaling pathway directly suppresses osteogenesis in OBs. Overactivation of OCs is dependent on RANKL secreted by senescent BMSCs, OBs, OCYs, and ADs. In addition, the deficiency of OCs-derived SPP1 inhibited the osteogenic differentiation of BMSCs. OCs-secreted ANG is a key factor preventing the senescence of neighboring ECs. Increased C-telopeptide in the aged bone matrix is thought to induce OC activation. The expression of Atg7 and Map1lc3a in OCYs is reduced in aged animals. Senescent OCYs-derived TPM1 was significantly reduced, thereby inhibiting the osteogenic differentiation of BMSCs. Senescent ADs-derived DPP4 also significantly inhibited osteogenesis. In addition, PCLAF, derived from bone marrow MACs in aged mice, binds to ADGRL2 receptors on ADs and promotes the senescence of ADs. Senescent immune cells secrete increased GCA, TGFβ1^+^CCR5^+^, and decreased IL-17, OSM, leading to bone loss. Elevated levels of immune cell-derived TNF-α, IL-8, and RANKL markedly promoted the activation of OCs. Senescent ECs exhibited decreased HIF/Notch signaling pathway and exo secretion, as well as increased miRNA-31-5p, accelerating bone loss. Decreased Cystatin-A secretion by aged skin keratinocytes leads to restricted OB differentiation and enhanced OC differentiation. FOXO3 in skeletal muscle cells plays a key role in counteracting aging-related cellular senescence. EP4 in neuronal cells regulates bone homeostasis and promotes bone regeneration by sensing prostaglandin E2 secreted by OBs. These aging-related cellular senescence leads to changes in bone cell function and fate, which ultimately contribute to the onset and development of OP. The red frames represent upregulated genes, and the blue frames represent down-regulated genes

The crosstalk between cells in the aging bone marrow microenvironment collectively determines the onset and progression of OP. Under aging conditions, BMSCs tend to undergo adipogenic differentiation, leading to reduced bone formation and increased bone marrow fat deposition. In the aging microenvironment, skeletal muscle cells, ECs, and MACs synergistically promote the senescence and adipogenic differentiation of BMSCs. The interaction between OBs and OCs is the core process of bone remodeling. Senescent OBs enhance the activation of OCs, thereby exacerbating bone loss. Additionally, OCs are excessively activated in the aging bone marrow environment because of factors secreted from OBs, OCYs, and ADs, resulting in a negative bone balance. The role of immune cells in the aging bone marrow cannot be overlooked. Senescent MACs, NEs, and B cells mediate the formation of OCs, whereas T cells reverse the adipogenic differentiation of BMSCs, influencing the balance between osteogenesis and adipogenesis. ECs exhibit dysfunction during aging, and a reduction in H-type vessels affects OB differentiation. Senescent ECs promote the adipogenic differentiation of BMSCs. In-depth research into the mechanisms of these cellular interactions will contribute to the development of precise intervention strategies for senile OP. The crosstalk between advancing age-related cellular senescence and OP pathogenesis is shown in Fig. 2 and Table 1.

**Table 1:** Role of cellular senescence and other age-related mechanisms in OP

Cell types	Molecules or signals	Variation	Function	References
BMSCs	Fgfr3	Down	Reduces colony-forming ability	^16^
	LepR	Up	Reduces osteogenic differentiation and induces OP	^16^
	CXCL12^+^ LepR^+^	Up	Towards a lipogenic cell lineage	^17,18^
	P62	Up	Induces cellular redox homeostasis, endoplasmic reticulum stress response, and inhibitory regulation of cell growth	^19^
	Prx1^+^Osx^−^	Down	Activation of apoptotic signaling pathways, reduction of mechanosensation	^20^
	SOX5	Down	Decreased HMGB2 enhancer activity, decreased H3K27ac and H3K4me1 levels	^21^
	TFEB	Down	Inhibition of autophagy	^22^
	DMNT-1/DNMT-3B	Down	Induces cellular senescence	^23–25^
	H3K4me1	Up	Induces histone methylation	^26^
	miRNA-34a-5p	Up	Induces senescence in BMSCs	^4^
	SASP	Up	Promotes the aged bone marrow microenvironment; facilitates OCs maturation and myeloid skewing of hematopoietic stem and progenitor cells	^5,30^
OBs	CD24^+^	Up	Induces significant growth arrest, SA-β-gal positivity, and osteogenesis restriction	^36^
	Osteolectin^+^	Down	Induces decreased bone formation, reduced lymphocytes	^37^
	mTORC1	Up	Increases Ca²^+^ influx and activates downstream NFAT/ATF3/p53 signaling	^38^
	METTL3	Down	Decreases Hspa1a mRNA stability	^39^
	ROS	Up	Activates FOXO pathway and blocks Wnt signal transduction	^40,41^
	IGF-I	Down	Inhibits OBs osteogenesis	^42^
	M-CSF/RANKL/WNT5A	Up	Promotes OCs formation	^43^
	SHN3	Up	Inhibits H-type blood vessel formation	^44^
	CD39-CD93-adenosine receptor	Down	Inhibits OBs osteogenesis	^45^
OCs	SPP1	Down	Inhibits osteogenic differentiation of BMSCs by reducing TGFβ1/SMAD3 signaling	^55^
	ANG	Up	Prevents senescence of neighboring ECs	^59^
	C-telopeptide	Up	Induces OCs activation	^60^
OCYs	SASP	Up	Accelerates bone loss	^65,66^
	Atg7/Map1lc3a	Down	Inhibits autophagy	^11^
	ROS	Up	Promotes cellular senescence	^67^
	RANKL/M-CSF	Up	Induces OCs activation	^73^
	TPM1	Down	Inhibits BMSCs osteogenesis	^74^
ADs	DPP4	Up	Inhibits osteogenesis	^17^
	RANKL	Up	Induces OCs activation	^77,78^
	SASP	Up	Promotes secondary senescence of ECs and OBs	^75^
	PCLAF	Up	Binds to the ADGRL2 receptor and promotes senescence	^79^
MACs/NEs	GCA	Up	Inhibits the osteogenic differentiation of BMSCs	^81^
MACs	OSM	Down	Inhibits the differentiation of BMSCs into the OBs lineage	^83^
NEs	TGFβ1^+^CCR5^+^	Up	Induces TRAF3 degradation via TGFβ1 and promotes bone loss	^84^
	RANKL/IL-8	Up	Induces OCs formation	^85^
Th1	TNF-α	Up	Induces OCs formation	^86^
Th17	IL-17	Up	Induces OCs formation	^87^
Treg	CD39-CD93-AdoR/ CTLA4	Up	Promotes osteogenic differentiation and inhibits OCs function	^45,88^
B lymphopoiesis	RANKL/G-CSF	Up	Promotes OCs activation	^89,90^
ECs	HIF/Notch	Down	Provides essential signals to the surrounding OBs	^91^
	miRNA-31-5p	Up	Promotes BMSC lipogenic differentiation by regulating the WNT pathway	^28^
	ECs-exo	Down	Upregulates ZBTB16 expression to promote BMSCs’ osteogenic differentiation	^95^

## Cellular senescence and other age-related mechanisms in osteoarthritis

OA is a disease that causes chronic pain in elderly individuals and accelerates disability worldwide.^96^ As a degenerative disease, aging-related cellular senescence has been confirmed to promote the progression of OA.^97^ Research data indicate that the incidence of OA increases with age.^98^ There is a significant increase in SA-β-Gal-positive cells, including chondrocytes, mesenchymal cells (MSCs), synovial cells (SYCs), ADs, and muscle cells, in vertebrate OA tissue, indicating that age-related significant cellular senescence occurs in OA.^99,100^ Senescent cells within joints continue to secrete SASP factors, further damaging the intra-articular structure.^101^ As research on OA continues to expand, the connection between senescent cells and OA has become increasingly clear. The various cellular senescence and other age-related mechanisms within osteoarticular joints in OA are reviewed below.

### Chondrocytes

In the early stages of arthritis, previously quiescent chondrocytes begin to proliferate, thus repairing damaged cartilage and preventing further tissue degradation. Unfortunately, newborn chondrocytes in this microenvironment are prone to entering a state of aging-related cellular senescence, leading to tissue degeneration and the onset of OA.^97,102^ Over time, chondrocytes in OA exhibit a more pronounced senescence phenotype and secrete SASP factors, such as matrix metalloproteinase (MMP)-13 and a disintegrin and metalloproteinase with thrombospondin motifs (ADAMTS)-5.^103^ Research has confirmed that the number of senescent chondrocytes is directly proportional to the severity of OA.^104^ Symptoms of OA develop after intra-articular injection of senescent chondrocytes into the joint cavity of healthy mice, suggesting that senescent chondrocytes are strongly associated with the development of OA.^105^ These data provide a rationale for senescent chondrocytes as drivers of OA.

Current studies reveal the crosstalk between OA and senescent chondrocytes. CircRREB1 is highly expressed in senescent chondrocytes and is involved in chondrocyte senescence and age-associated OA by regulating fatty acid synthase-related lipid metabolism.^106^ Research has shown that PDZ domain containing 1 (PDZK1) belongs to the Na^+^/H^+^ exchange regulatory factor family.^107^ Mechanical overload leads to a reduction in PDZK1, which has been observed in the articular cartilage of OA patients, elderly mice, and OA mice. Deficiency of PDZK1 is a key factor in the progression of OA, where biomechanical induction leads to mitochondrial dysfunction and chondrocyte aging.^107^ Additionally, SIRT6 was found to be significantly down-regulated in the cartilage tissues of OA patients, which promotes chondrocyte senescence by up-regulating senescence markers via the SIRT6-STAT5 signaling pathway.^108^ One study revealed that ADAM19 is a key senescence-related gene in cartilage and that lowering ADAM19 expression significantly reduces the senescent phenotype of chondrocytes.^109^ The expression of senescence markers is significantly increased in chondrocytes from OA patients, and further mechanistic studies revealed that myosin light chain 3 (MYL3) deficiency in chondrocytes promotes clathrin-mediated endocytosis and Notch signaling, leading to chondrocyte senescence and OA.^110^

Senescent chondrocytes also support the formation of an intra-articular inflammatory microenvironment through the secretion of SASP, which mediates crosstalk between senescent chondrocytes and neighboring cells and ultimately promotes neighboring cell senescence.^111,112^ In addition to the SASP, senescent chondrocytes secrete additional EVs to trigger senescence in their neighboring cells.^113^ These data confirmed that the production of EVs in senescent chondrocytes is positively correlated with the number of senescent cells. Mechanistically, EVs affect neighboring cell senescence by regulating the expression of miR-34a, miR-24, and miR-150.^113^ Recent crosstalk between extracellular matrix (ECM) and senescent chondrocytes has also been revealed. ECM sclerosis downregulates histone deacetylase 3 (HDAC3), which contributes to Parkin acetylation, which in turn activates mitochondrial autophagy, leading to a senescent phenotype in chondrocytes and accelerated OA symptoms.^114^ Further explorations are needed to reverse chondrocyte senescence to maintain the integrity of the articular cartilage.

### Mesenchymal cells

The MSCs that primarily influence joint structure are mainly subchondral bone marrow MSCs, whose depletion and abnormal repair accelerate pathological changes in OA.^115^ Dysfunction of RNA-binding proteins is closely associated with aging-related cellular senescence and OA. An exploration of the interaction of the RNA-binding protein PUM1 with the 3ʹ-UTR of Toll-like receptor 4 (TLR4) and its effect on NF-κB activity in MSCs revealed that the PUM1 protein alleviated MSCs senescence and OA progression by inhibiting TLR4-mediated NF-κB activation.^116^ Moreover, RNA modification is involved in the regulation of aging-related cellular senescence. Elevated m6A levels and reduced expression of the m6A demethylase ALKBH5 were detected in senescent MSCs. Downregulation of ALKBH5 expression promoted MSC senescence and OA by enhancing m6A modification to increase the stability of CYP1B1 mRNA and induce mitochondrial dysfunction.^117^ A study also demonstrated that MSC senescence and OA injury could be reversed by rescuing the levels of the heterochromatin-associated protein HP1α and the nuclear lamina protein LAP2, promoting cell division and inhibiting aging-associated inflammation.^118^

Crosstalk between chondrocytes and subchondral MSCs is also closely linked to the development of OA. By co-culturing senescent subchondral bone marrow MSCs with chondrocytes from OA patients in vitro, chondrocytes were found to still exhibit a catabolic phenotype.^119^ The same conclusion was also confirmed in vivo. After intra-articular injection of senescent subchondral bone marrow MSCs into the joints of young male mice, histological analysis revealed a significant increase in articular cartilage decomposition, and micro-CT analysis revealed a significant reduction in subchondral bone volume. These data suggest that senescent subchondral bone marrow MSCs destroy cartilage and joints.^119^ Cao et al. noted that senescent chondrocytes restrict the multipotency of subchondral bone marrow MSCs, whereas MSCs accelerate the apoptosis of senescent chondrocytes, which leads to OA via crosstalk.^120^ Furthermore, the immunomodulatory properties of MSCs have been demonstrated to significantly influence the progression of OA. The synthesis and catabolism of metabolic factors are regulated by cytokines secreted by MSCs, which promote the secretion of anti-inflammatory factors by SYCs.^121^

The aged skeletal stem cells in the cartilage have been reported to potentially promote OA. Murphy et al. reported a significant decrease in the number and secretory function of skeletal stem cells in the cartilage of elderly animals compared with young animals, indicating that aged skeletal stem cells contribute to cartilage pathology in OA.^122^ Interestingly, a special type of skeletal stem cell, Gremlin 1^+^ cells, in joint cartilage potentially promotes OA formation.^123,124^ Gremlin 1^+^ cells have been demonstrated to obtain the capacity to differentiate into OBs, chondrocytes, and reticular BMSCs. A gradual loss of Gremlin 1^+^ cells was observed in the joints of the aged mice, which resulted in the progression of OA. This study confirmed that the absence of Gremlin 1^+^ cells may lead to OA progression and that OA progression can be reversed by developing therapeutic regimens that target Gremlin 1^+^ cells.^124^ Overall, altering the differentiation potential and secretion behavior of aged subchondral bone marrow MSCs and skeletal stem cells may offer a promising avenue for OA treatment.

### Synovial cells

SYCs primarily include synovial fibroblasts and synovial MACs, which are responsible for lubricating joints and maintaining microenvironment homeostasis.^125^ While the role of the synovium in OA has gradually been recognized, the relationship between senescent SYCs and OA remains unclear.

Studies have indicated that during arthritis inflammation, synovial tissue from mice stained with SA-β-gal presents visibly senescent cells, although the specific cell types involved are not clearly identified.^99^ Research has revealed a significant presence of P16-expressing MACs around aged tissues, suggesting that the infiltration of senescent MACs may contribute to pathological changes in joint tissues.^126^ However, the impact of the secretory function of senescent MACs in OA remains unclear. It has been suggested that synovial MACs deplete the cell surface molecule CD38 by secreting nicotinamide adenine dinucleotide (NAD), thereby accelerating senescence.^127^ Concurrently, the SASP derived from senescent cells also induces aging in synovial MACs. Additionally, synovial fibroblasts in elderly OA exhibit decreased synovial fluid secretion and increased levels of pro-inflammatory mediators, leading to cartilage degradation and joint damage.^128^ Synovial fibroblasts in aged OA patients also accelerate cartilage deterioration by activating the PI3K and ERK pathways through the secretion of adipokines.^129^ Consequently, while the relationship between senescent synoviocytes and age-related OA remains to be elucidated, crosstalk between these cells and bone cells appears to influence the progression of age-related OA.

### Subchondral bone cells

In addition to the pivotal role of chondrocytes in the development of OA, the impact of subchondral bone cells has also garnered widespread attention in recent years. Compared with that in young joints, the microenvironment of the subchondral bone in elderly patients with OA significantly changes. Subchondral senescent pre-OCs with a unique secretory phenotype have been identified in the early stages of OA.^130^ At the molecular level, senescent pre-OCs enhance the osteogenic differentiation of BMSCs by activating the cyclooxygenase 2 (COX2)-prostaglandin E2 (PGE2) pathway.^130^ The bone resorption activity of subchondral OCs leads to a sharp increase in the activity of TGF-β1 in the subchondral bone cells of OA patients.^131^ Increased TGF-β1 activates the Smad2/3 signaling pathway, attracting bone progenitor cells to the area of bone remodeling and promoting the formation of abnormal bone tissue.^131^ Furthermore, subchondral OCs in natural senescence-induced OA ubiquitinated HIF-1α in chondrocytes by inducing H-type vasculature to elevate the oxygen content, leading to chondrocyte senescence.^132^ The knockdown of lymphocyte cytoplasmic protein 1 (Lcp1) maintains the hypoxic state of the subchondral bone microenvironment by disrupting angiogenesis and delaying pathological changes in OA.^132^ Moreover, senescent OCs in the subchondral bone microenvironment also promote the formation of sensory nerves by secreting Netrin-1.^133^ Additionally, RANKL and MMP-9 derived from H-type vessels induce the migration and maturation of OCs.^134^ With aging, the ratio of RANKL to OPG in subchondral OCYs increases, thereby promoting the activation of OCs.^135^ Various factors are released into subchondral OBs, such as PGE2, interleukin-6 (IL-6), matrix metallopeptidase 9, vascular endothelial growth factor (VEGF), and RANKL, further promoting the differentiation of OCs and the erosion of subchondral bone.^133^ Importantly, after the use of the INK-ATTAC “suicide” transgene, “senolytic” compounds, or JAK inhibitors to remove or inhibit SASP secretion from aged osteogenic lineage cells, elderly mice exhibited improved bone microstructure and reduced OA.^12^ These mechanisms act on subchondral bone cells to exacerbate the pathological process of OA. Inhibiting the senescence of subchondral bone cells may be an effective treatment for OA.

### Other cells

Individuals with a body mass index exceeding 28 have a high incidence of OA, as increased mechanical loading is a key factor driving OA occurrence.^100^ Recent studies have shown that senescent ADs in joints may be a significant source of inflammatory mediators in OA.^136^ Similar to other senescent cells, senescent ADs secrete SASP factors that accelerate the aging of other cells and the progression of OA.^75^ Additionally, OA patients commonly experience muscle function impairments. Studies indicate that the regeneration of senescent musculoskeletal cells is inhibited by aging satellite cells. In 24-month-old mice, satellite cells enter an aging state by unblocking p16. Senescent satellite cells significantly inhibited the regeneration of musculoskeletal cells, suggesting that senescent satellite cells and muscle cells may also be involved in the development of OA.^137^

**Fig. 3:**
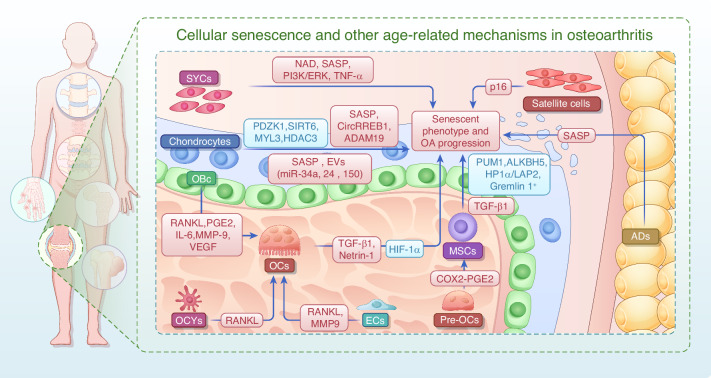
Cellular senescence and other age-related mechanisms in the knee joint lead to OA progression. Senescent chondrocytes promote the expression of the senescent phenotype by up-regulating SASP, CircRREB1, and ADAM19 and down-regulating PDZK1, SIRT6, and MYL3. Meanwhile, senescent chondrocytes also promote senescence in neighboring cells by secreting SASP and EVs (containing miR-34a, miR-24, and miR-150). In addition, ECM sclerosis downregulates HDAC3 and activates mitochondrial autophagy, leading to a chondrocyte senescence phenotype and accelerated OA symptoms. Depletion and aberrant repair of MSCs accelerate pathological changes in OA. Senescent MSCs presented decreased expression of PUM1, ALKBH5, and HP1α/LAP2. Senescent chondrocytes limit the pluripotency of MSCs, while MSCs accelerate the apoptosis of senescent chondrocytes. The absence of Gremlin 1^+^ cells may contribute to the progression of OA. Senescent SYCs accelerate senescence and cartilage damage by increasing NAD, SASP, PI3K/ERK, and TNF-α levels. Changes in subchondral bone cells with senescence in OA also promote OA progression. OCs accelerate intra-articular senescence and OA pathology by regulating COX2/PGE2, TGF-β1, HIF-1α, Lcp1, and Netrin-1. ECs, subchondral OCYs, and subchondral OBs activate OCs via RANKL and accelerate subchondral bone erosion. OBs release PGE2, IL-6, MMP-9, and VEGF to further promote OCs differentiation. Senescent ADs secrete SASP factors, accelerating the senescence of other cells and the progression of OA. Senescent satellite cells significantly inhibit musculoskeletal cell regeneration by unblocking p16. All of these factors contribute to the malignant crosstalk of senescence in OA and accelerate its progression. The red frames represent upregulated genes, and the blue frames represent down-regulated genes

The onset of OA is driven by complex crosstalk among multiple cell types. In the early stages of OA, chondrocytes proliferate to repair damage, but with the advancement of age, they secrete SASP factors that degrade the cartilage matrix and induce inflammation. The depletion of MSCs accelerates OA progression. Senescent MSCs inhibit chondrocyte proliferation while promoting their senescence, creating a vicious cycle. Senescent SYCs reduce the secretion of synovial fluid and release pro-inflammatory factors that exacerbate inflammation. Subchondral bone cells promote OC activity through factors such as RANKL. Abnormal H-type vessels accelerate chondrocyte senescence. Overactivated OCs enhance bone resorption and secrete factors that promote osteogenesis in BMSCs and remodeling of subchondral bone, further aggravating OA pathological progression. ADs secrete SASP to induce senescence in neighboring cells, and the senescence of satellite cells inhibits muscle regeneration, affecting joint stability. Intervention strategies targeting intercellular crosstalk may provide new directions for OA treatment. Molecules or signals involved in cellular senescence and other age-related mechanisms during OA are shown in Fig. 3 and Table 2.

**Table 2:** Signals involved in cellular senescence and other age-related mechanisms during OA

Cell types	Molecules or signals	Variation	Effect	References
Chondrocytes	SASP	Up	Promotes senescence phenotype	^103^
	CircRREB1	Up	Promotes chondrocyte senescence by regulating fatty acid synthase-related lipid metabolism	^106^
	PDZK1	Down	Promotes mitochondrial disorders and chondrocyte aging	^107^
	SIRT6	Down	Upregulates senescence markers via the SIRT6-STAT5 signaling pathway	^108^
	ADAM19	Up	Promotes the senescence phenotype	^109^
	MYL3	Down	Promotes clathrin-mediated endocytosis and Notch signaling	^110^
	miR-34a/miR-24/miR-150	Up	Affects the senescence of neighboring cells	^113^
	HDAC3	Down	Contributes to Parkin acetylation and activates mitochondrial autophagy	^114^
MSCs	PUM1	Down	Activates TLR4-mediated NF-κB activation	^116^
	ALKBH5	Down	Enhances m6A modification to enhance the stability of CYP1B1 mRNA and induce mitochondrial dysfunction	^117^
	HP1α/LAP2	Down	Promotes aging-associated inflammation	^115^
	Gremlin 1^+^	Down	Decreases in quantity and secretory function and contributes to the cartilage pathology of OA	^124^
SYCs	NAD	Up	Accelerates CD38 depletion	^127^
	SASP	Up	Induces senescence phenotype	^128^
	PI3K/ERK	Up	Accelerates cartilage deterioration	^129^
Subchondral pre-OCs	COX2/PGE2	Up	Enhances osteogenic differentiation of BMSCs	^130^
Subchondral OCs	TGF-β1	Up	Activates the Smad2/3 signaling	^131^
	HIF-1α	Down	Increases oxygen levels	^132^
	Lcp1	Up	Promotes angiogenesis	^132^
	Netrin-1	Up	Promotes sensory nerve formation and low back pain	^133^
ECs	RANKL/MMP-9	Up	Induces the migration and maturation of OCs	^134^
Subchondral OCYs	RANKL	Up	Promotes the activation of OCs	^135^
Subchondral OBs	PGE2/IL-6/MMP-9/VEGF/RANKL	Up	Promotes the differentiation of OCs and the erosion of subchondral bone	^133^
ADs	SASP	Up	Accelerates the aging of other cells	^75^
Satellite cells	p16	Up	Inhibits the regeneration of musculoskeletal cells	^137^

## Cellular senescence and other age-related mechanisms in intervertebral disc degeneration

The intervertebral disc (IVD) is composed of the inner nucleus pulposus (NP) and the outer annulus fibrosus (AF). The incorporation of the upper and lower vertebral endplates creates a relatively enclosed microenvironment within the IVD.^138^ Increased expression of aging-associated cell phenotypes has been detected in IVD within elderly vertebrates.^139–141^ The accumulation of aging-related cellular senescence in the IVD provides a new research direction for IVD development.^142–146^ Initially, aging prevents the proliferation and formation of new IVD cells. Subsequently, aging-related cellular senescence to the senescent state results in increased SASP secretion, which accelerates inflammation and degradation of the disc matrix.^147,148^ In conclusion, aging promotes the transition of the IVD microenvironment to an inflammatory microenvironment by activating multiple pathways. Precisely exploring the mechanisms underlying cellular senescence and other age-related mechanisms holds promise for reversing IVD.

### Nucleus pulposus cells

As the main effector cells, nucleus pulposus cells (NPCs) are responsible for ECM synthesis and regulate homeostasis within the IVD.^149^ NPCs accelerate the production of tissue growth factors such as connective tissue growth factor (CTGF/CCN2) by secreting TGF-β. Moreover, CTGF/CCN2 promotes the proliferation of NPCs.^150^

As age increases, NPCs exhibit reduced CTGF/CCN2 and increased SASP secretion, leading to increased matrix metalloproteinases (MMP3 and MMP7), which results in ECM breakdown.^151^ Studies have shown that abnormal mechanical stress is a primary driving factor for NPCs aging. Piezo1, a mechanosensitive ion channel, links mechanical stimuli to signal transduction.^152^ Increased Piezo1 was observed in NPCs from excessive stress or elderly individuals, leading to mitochondrial dysfunction and inflammatory infiltration and triggering senescence in NPCs.^153^ Furthermore, accumulated ROS in aged IVD accelerates NPCs aging through ROS-mediated biological signaling.^154^ Interestingly, senescent NPCs are also associated with the epigenetic regulation of non-coding RNAs. Upregulation of miR-182-5p and downregulation of circERCC2 are found in the NPCs of elderly individuals. circERCC2 regulates NPCs aging through the miR-182-5p/SIRT1 cascade.^155^ The Keap1/Nrf2/Wnt5a axis was identified as a novel signaling pathway that protects NPCs from senescence.^156^ In addition, aberrant genomic DNA damage promoted inflammatory senescence in NPCs through activation of the cyclic GMP-AMP synthase/IFN gene-stimulating factor (cGAS/STING) axis but not melanoma absent 2 (AIM2) inflammasome assembly.^157^ ROS accumulation and mitochondrial dysfunction were observed in degenerating NP tissues from rats and young adults, which caused the senescence of NPCs. Senescent NPCs promote disc degeneration through the p53‒p21 signaling axis, as confirmed by a series of experiments, including RNA-seq.^142^ Sustained oxidative stress in the IVD also leads to impaired antioxidant systems in NP cells. Transcriptome sequencing revealed that the antioxidant glutaredoxin3 (GLRX3) is significantly reduced in senescent NPCs and that dysregulation of GLRX3 disrupts redox homeostasis and contributes to NPCs’ senescence and disc degeneration.^158^ Notably, senescence of NPCs has been shown to be closely related to autophagy. NLR family member X1 (NLRX1) deficiency in NPCs exhibits mitochondrial rupture and excessive mitochondrial autophagy, which in turn leads to cellular senescence and disc degeneration.^159^ Another study confirmed that the inflammatory factor TNF-mediated senescence of NPCs involves chaperone-mediated autophagy (CMA) inhibition.^160^ Sequencing analyses revealed that CMA deficiency led to the accumulation of phospholipase C gamma 1 (PLCG1), which triggered calcium overload-induced cellular senescence in NPCs. These data indicate that senescent NPCs expedite the process of IVDD, providing new therapeutic approaches for rescuing aging NPCs and combating IVDD.

### Annulus fibrosus cells

Research has revealed significant heterogeneity in external and internal AF cells (AFCs).^138^ External AFCs are spindle-shaped and rich in fibroblasts and type I collagen. The internal AFCs are more rounded and enriched in chondrocytes and type II collagen. Healthy AFCs exhibit a sensitive stress response and strong tensile properties that effectively limit the prominence of NP.^161^ Sonic hedgehog hormone (SHH) from NPCs has been shown to promote the survival and normal differentiation of AFCs.^162^ With age, the SHH pathway is gradually inhibited in NPCs.^163,164^ The absence of SHH in NPCs has been demonstrated to result in AFCs losing their polarity and characteristic hierarchical structure.^162^ This change may greatly reduce the tensile strength of the AFCs and the matrix degradation in the AF, which ultimately fails to prevent NP protrusion.

Recent studies have found that activation of the Keap1/Nrf2/Wnt5a signaling axis inhibits senescence and extracellular matrix breakdown in AFCs, thereby effectively delaying natural aging-induced disc degeneration.^156^ Furthermore, studies have confirmed that sites of AF injury in IVDD exhibit an abnormal accumulation of ROS and a significantly reduced number of AFCs, thus limiting AF regeneration.^165^ Observations of human AF tissue have shown that the AF/chondral endplate border is adherent to CD133^+^ stem cells that are committed to either the chondrogenic or adipogenic spectrum.^166^ A significant loss of the stem cell pluripotency markers Notch1, Jagged1, C-KIT, and CD166 was found in the AF of aged rabbits, which may contribute to disc aging.^167^ Inhibiting the aging of AFCs may provide reliable information for IVD regeneration.

### Cartilaginous endplate cells

Cartilage endplate cells (CEPCs), located between the IVD and vertebrae, not only serve a transitional function but also support the healthy growth of IVD cells.^168^ Similar to cartilage cells, CEPCs are responsible for ECM synthesis. These cells restrict the entry of NPCs into the vertebral body and alleviate the hydrostatic pressure generated by mechanical loads.^169^

Senescence of CEPCs has also been observed in aged IVDs. Decreased proteoglycan secretion and increased type X collagen secretion in CEPCs of aged individuals lead to ECM calcification.^170,171^ It has been shown that the permeability of the endplate is attributed to the presence of a microvascular network within the endplate. Various nutrients of different sizes and charges maintain the health of IVD cells through the cartilage endplate.^172,173^ However, calcified CEPCs exhibit reduced permeability, limiting the absorption of nutrients and oxygen and slowing the clearance of harmful metabolites to disrupt IVD homeostasis.^138^ Cao et al. reported that the number of p16^+^ TRAP^+^ OCs significantly increased in the endplates of aged mice, while the endplates degenerated. These OCs induce sensory nerves to innervate the porotic endplates by mediating the secretion of Netrin-1 and nerve growth factor (NGF), which suggests that the senescent OCs in the endplates of aged mice are closely related to degenerative changes in the endplates and the development of low back pain.^174^ Ni et al. reported that Trap^+^ OCs secrete the neurotransmitter Netrin-1, which mediates the gradual growth of sensory nerves into aged endplate tissue. Furthermore, they also revealed that significantly elevated levels of PGE2 in aged endplate tissue stimulated EP4 receptors on sensory nerves and activated the downstream PKA/CREB signaling pathway, which mediated the inward flow of Na ions, thereby leading to pain signaling.^175^ Meanwhile, Chen et al. revealed a new mechanism of OCs in endplate remodeling. The abnormal stress induced by lumbar spine instability activates Hippo signaling, which initiates cartilage endplate remodeling through activation of the OCs differentiation gene CCL3. Inhibition of Hippo signaling activation or resupply of YAP in CEPCs effectively prevents their transformation into cheese-like endplates and subsequent lumbar degeneration.^146^ Furthermore, Ni et al. discovered that senescent MACs accumulated in sclerotic endplates in an aged male mouse model. Senescent-like MACs affect immunovascular communication by secreting the pro-angiogenic cytokine IL-10, which leads to endplate sclerosis by affecting signal transduction and activator of transcription 3 (STAT3) signaling pathways.^176^ Measures to rescue senescent CEPCs are indispensable for the treatment of IVDD.

**Fig. 4:**
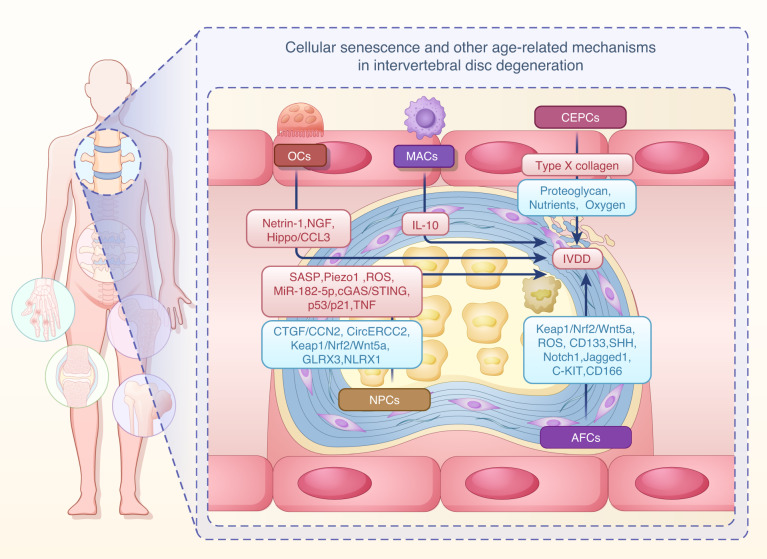
Cellular senescence and other age-related mechanisms in IVD accelerate IVDD. With age, NPCs lead to ECM breakdown through decreased CTGF/CCN2 and increased SASP secretion. An increase in Piezo1 and a decrease in circERCC2 were also observed in senescent NPCs. Blockade of the Keap1/Nrf2/Wnt5a axis or activation of the cGAS/STING axis promoted the senescence of NPCs. Furthermore, ROS accumulation, p53/p21 activation, GLRX3 dysregulation, a lack of NLRX1, and TNF infiltration also contributed to the senescence of NPCs. Decreased SHH secretion in senescent NPCs leads to loss of polarity and the characteristic hierarchical structure of AFCs. ROS accumulation and deletion of Keap1/Nrf2/Wnt5a, CD133^+^, Notch1, Jagged1, C-KIT, and CD166 trigger senescence in AFCs. Decreased proteoglycan secretion and increased X-type collagen secretion in CEPCs led to calcification of the ECM. Calcified CEPCs limit nutrient and oxygen uptake, thereby disrupting IVD homeostasis. Senescent OCs induce pain via Netrin-1 by inducing sensory nerves into the aging endplate. Abnormal stress activates Hippo signaling to initiate cartilage endplate remodeling. Senescent macrophages induce endplate sclerosis by secreting IL-10. These factors ultimately contribute to the cellular changes associated with aging and the development of IVDD. The red frames represent upregulated genes, and the blue frames represent down-regulated genes

IVDD is driven by a complex interplay of NPCs, AFCs, CEPCs, OCs, and immune cells. NPCs maintain ECM production, but as they age, their SASP increases and promotes matrix degradation. Moreover, mechanical stress, ROS accumulation, and non-coding RNA dysregulation accelerate the aging of NPCs and trigger IVDD. The AFCs depend on SHH secreted by NPCs to maintain and lose polarity with decreasing SHH, which reduces AF strength. In addition, excessive accumulation of ROS and loss of stem cell markers reduced the regenerative ability of AFCs, exacerbating IVD degeneration. Senescence of CEPCs leads to calcification of the ECM, reducing endplate permeability and disrupting IVD homeostasis. Senescent OCs promote endplate remodeling and secrete Netrin-1 and PGE2, which induce nerve invasion and cause pain. MACs accumulate and secrete IL-10 to promote endplate sclerosis and exacerbate IVD degeneration. In conclusion, IVDD is driven by multiple cellular crosstalk, and interventions targeting key cellular interactions may become a new therapeutic strategy in the future. The mechanisms linking cellular senescence and other age-related changes to IVDD are shown in Table 3 and Fig. 4.

**Table 3:** Cellular senescence and other age-related mechanisms in IVDD

Cell types	Molecules or signals	Variation	Effect	References
NPCs	SASP	Up	Accelerates inflammation and degradation of the disc matrix	^147,148^
	CTGF/CCN2	Down	Increases matrix metalloproteinases and ECM breakdown	^150,151^
	Piezo1	Up	Promotes mitochondrial dysfunction and inflammatory infiltration	^153^
	CircERCC2	Down	Regulates NPCs’ aging through the miR-182-5p/SIRT1 cascade	^155^
	Keap1/Nrf2/Wnt5a	Down	Protects NPCs from senescence	^156^
	cGAS/STING	Up	Promotes inflammation and cellular senescence	^157^
	ROS	Up	Promotes mitochondrial dysfunction	^142^
	p53/p21	Up	Promotes disc degeneration	^142^
	GLRX3	Down	Disrupts redox homeostasis and contributes to cellular senescence	^158^
	NLRX1	Down	Promotes mitochondrial rupture and excessive mitochondrial autophagy	^159^
	TNF	Up	Triggers calcium overload-induced cellular senescence	^160^
	SHH	Down	Promotes AFCs losing their polarity and characteristic hierarchical structure	^162^
AFCs	Keap1/Nrf2/Wnt5a	Down	Promotes senescence and extracellular matrix breakdown	^156^
	ROS	Up	Promotes cellular senescence	^165^
	CD133/Notch1/Jagged1/C-KIT/CD166	Down	Reduces stem cell pluripotency markers	^166,167^
CEPCs	Proteoglycan	Down	Accelerates ECM calcification	^170^
	Type X collagen	Up	Accelerates ECM calcification	^171^
	Nutrients/Oxygen	Down	Accumulates harmful metabolites	^138^
OCs	Netrin-1	Up	Mediates the gradual growth of sensory nerves into aged endplate tissue	^174,175^
	PGE2	Up	Activates PKA/CREB and induces low back pain in elderly individuals	^175^
	Hippo	Up	Initiates cartilage endplate remodeling through activation of the OCs differentiation gene CCL3	^146^
MACs	IL-10	Up	Leads to endplate sclerosis by affecting STAT3 signaling pathways	^176^

## Cellular senescence and other age-related mechanisms in other bone disorders

### Cellular senescence and other age-related mechanisms in rheumatoid arthritis

Rheumatoid arthritis (RA) is characterized by chronic inflammatory infiltration that severely impairs joint function.^177^ Studies indicate that aging promotes the onset and progression of RA. Clinical data confirm that the incidence of RA in elderly individuals is ~14 times greater than that in younger individuals and that later onset corresponds to more severe loss of joint function.^178,179^ Premature immune senescence, including expansion of late-differentiated T cell proliferation, increased SASP secretion, and shortened telomeres, has been reported in RA patients.^180^ Late-differentiated T cells are defined as CD4^+^ and CD8^+^CD28^−^ T cells, which are detected in both elderly and RA patients.^181,182^ Importantly, the number of late-differentiated T cells is proportional to the severity of RA.^183^ Additionally, senescent synovial fibroblasts in RA exhibit increased SASP secretion, further exacerbating RA.^184^ Recent studies have also shown that telomere shortening in senescent cells promotes the extra-articular manifestations of RA.^185^ Telomere shortening also triggers enhanced autoantigen reactivity, indicating that cellular senescence is one of the risk factors for RA.^186^ In summary, the senescence of immune cells and synovial fibroblasts exacerbates the symptoms of RA, and rejuvenating senescent cells may hold significant value in RA.

### Cellular senescence and other age-related mechanisms in bone tumors

Growing data have supported the contradictory dual role of senescent cells in bone tumors.^187,188^ On the one hand, dysfunction of senescent cells promotes tumorigenesis. Studies have revealed the abnormal accumulation of senescent immune cells in the osteosarcoma of elderly patients.^189^ Senescent immune cells exhibit weak antigen presentation, reduced antibody production, and low cytotoxic activity, thereby promoting tumor cell evasion.^190,191^ Senescent immune cells fail to proliferate and exhibit a weakened response to antigen stimulation in tumors.^192^ Moreover, the accumulation of senescent immune cells in osteosarcoma leads to increased secretion of SASP components. An increased SASP in the tumor microenvironment is linked to chronic inflammation, angiogenesis, mitosis, migration, and invasion, thereby supporting tumor cell proliferation and migration.^193,194^ Known SASP factors, such as IL-6, IL-8, and CCL5, are key mediators driving tumor cell proliferation.^195,196^ VEGF and CXCL5 have been shown to promote angiogenesis in tumors.^197,198^ The MMP contributes to tumor migration by degrading the ECM.^199^ Additionally, senescent immune cells exhibit attenuated bone tumor cell attack due to poor memory function.^200,201^

On the other hand, cell cycle arrest in senescent cells limits tumor development. The senescent cells induced by p16 and p21 exhibit irreversible growth arrest, directly inhibiting tumor cell proliferation. Several components of the senescent cell-derived SASP restrict tumor cell growth. IGF binding protein 7 and GROα in the SASP have been found to promote tumor cell growth arrest.^202,203^ Plasminogen activator inhibitor 1 also enhances replicative senescence in tumor cells.^204^ Thus, the induction of tumor cell senescence has a significant effect on anti-bone tumor therapy. Indeed, the accumulation of senescent cells has been observed in tumor specimens following the administration of chemotherapy, radiotherapy, or targeted therapy.^205–207^ This is closely related to the severe DNA damage triggered by anti-tumor therapies. Notably, the threshold for the therapeutic induction of tumor cell senescence requires further definition to reduce the occurrence of this process in non-tumor cells.^187^ Further studies are needed to document the behavior of various senescent cells in bone tumors to gain a more comprehensive understanding of their dual role in patients with bone tumors.

### Cellular senescence and other age-related mechanisms in ankylosing spondylitis

Currently, there is a lack of research revealing the relationship between aging-induced cellular senescence and ankylosing spondylitis (AS). However, some clues can be obtained from the clinical manifestations of elderly AS patients. Elderly individuals always exhibit reduced function of various types of cells combined with specific lesions of AS, increasing the susceptibility of elderly AS patients to severe complications. The current observation that elderly AS patients exhibit severe OP, rapid sarcopenia, and an increased incidence of cardiovascular events indicates that aging-related cellular senescence is involved in the aggravation of AS.^208^ It is well known that pathological new bone formation accelerates bone fusion in AS. Recent studies have indicated that chondrocytes promote ectopic new bone formation in AS through the BMP6/pSmad1/5 pathway, suggesting that inducing chondrocyte senescence in AS may help alleviate its progression.^209^ Future research on the role of cellular senescence and other age-related mechanisms in the occurrence and development of AS is worthy of attention.

## Cellular senescence and other age-related mechanisms in “bone‒organ” disorders

In addition to cells within the bone marrow, cells in other organs play pivotal roles in aging-related bone disease. Studies have shown that premature skin aging leads to bone loss in mice.^210^ Cystatin-A, which is secreted by skin keratinocytes, binds to receptors for activated C-kinase 1 in OBs and OCs progenitor cells, resulting in enhanced OBs differentiation and restricted OCs differentiation. This study connects skin aging with age-related bone loss and confirms that improving the level of Cystatin-A in the skin could serve as a potential topical treatment for age-related OP.^210^ The same research group also generated a single-cell nuclear transcriptome atlas of skeletal muscle, revealing the critical role of FOXO3 in counteracting skeletal muscle aging.^211^ These findings provide a theoretical basis for further development of diagnostic and intervention strategies for skeletal muscle aging. Senescent skeletal muscle cells secrete fewer myogenic factors, which impairs the maintenance of skeletal homeostasis.^211^ Neuronal cells have also been proven to regulate bone homeostasis.^212^ PGE2 receptor 4 in neuronal cells regulates bone homeostasis and promotes regeneration by sensing PGE2 secreted by OBs. Aging-induced hypofunction of neuronal cells may affect bone regeneration through reduced receptor expression. Moreover, the abnormal elevation of OB-derived sclerostin associated with aging dysregulates the Wnt/β-catenin pathway in the brain and increases the production of β-amyloid through the β-catenin-BACE1 signaling pathway, thereby accelerating cognitive impairment.^213^ This study reveals the mechanism of “bone‒brain” interactions and provides a new strategy for the treatment of Alzheimer’s disease. This study demonstrated that the expression of Sirtuin 2 (SIRT2) was increased in the hepatocytes of aged individuals. Liver-specific SIRT2 deletion upregulated the transfer of hepatic leucine α-2-glycoprotein 1 (LRG1) to bone marrow mononuclear cells, which inhibited the nuclear translocation of NF-κB p65 and the activation of OCs, ultimately slowing the progression of OP in elderly individuals.^214^ The discovery of the “bone‒liver” axis provides a new perspective for the treatment of OP.

Aging is usually accompanied by disruption of gut barrier integrity, changes in the composition of the gut microbiota, and increased susceptibility to a variety of aging-related diseases.^215^ An oral hydrogel microsphere system has been developed to achieve targeted delivery of BMSCs by adhering to intestinal epithelial cells.^216^ Following 8 weeks of continuous oral treatment, a significant increase in bone density and trabecular number and a decrease in trabecular spacing were observed in aged mice.^216^ RNA sequencing revealed that the system effectively reversed mitochondrial senescence in BMSCs by activating the AMPK-SIRT1 pathway and promoted regeneration of aged bone tissue.^216^ Furthermore, the probiotic strain Lactobacillus plantarum TWK10 has been found to attenuate aging-related bone loss by modulating intestinal dysbiosis and increasing total short-chain fatty acid levels.^217^ It has been reported that gut microbial tryptophan metabolites promote osteogenesis and inhibit OCs activation by enhancing the production of IL-10 by M2 MACs in the intestinal lamina propria into the bone marrow.^218^ Collectively, these data support the inextricable link between the “bone-gut” axis and suggest that the “bone-gut” axis holds promise for the treatment of OP in the elderly. Owing to the inadequacy of existing experimental techniques, the mechanism of the complex crosstalk between bone and various organs remains obscure. It is believed that advanced technologies, such as bone microarrays, organoids, and assemblies, will be able to elucidate the bone‒organ communication mechanism in a more systematic way in the future, thus contributing to the treatment of anti-bone aging diseases. The cellular senescence and other age-related mechanisms in “bone‒organ” disorders are shown in Table 4.

**Table 4:** Cellular senescence and other age-related mechanisms in “bone-organ” disorders

Organ	Molecules or signals	Variation	Effect	References
Skin	Cystatin-A	Down	Inhibits osteogenesis, promotes OCs activation	^210^
Skeletal muscle	FOXO3	Down	Inhibits myogenic factors and impairs the maintenance of skeletal homeostasis	^211^
Nerve	EP4	Down	Regulates bone homeostasis and promotes regeneration by sensing prostaglandin E2 secreted by OBs	^212^
Brain	β-amyloid	Up	Elevates OB-derived sclerostin associated with aging increases the production of β-amyloid in the brain and accelerating cognitive impairment	^213^
Liver	SIRT2	Up	Downregulates the transfer of LRG1 to bone marrow mononuclear cells, inhibits nuclear translocation of NF-κB p65, activates OCs, and promotes the progression of OP	^214^
Gut	AMPK-SIRT1	Down	Increases mitochondrial senescence in BMSCs	^216^
	TWK10	Down	Increases aging-related bone loss by modulating intestinal dysbiosis and reduces total short-chain fatty acid levels	^217^
	Microbial tryptophan metabolites	Down	Inhibits osteogenesis and promotes OCs activation by reducing the production of IL-10 by M2 macrophages in the intestinal lamina propria into the bone marrow	^218^

## Perspective and translation of the cellular senescence and other age-related mechanisms in bone diseases

**Fig. 5:**
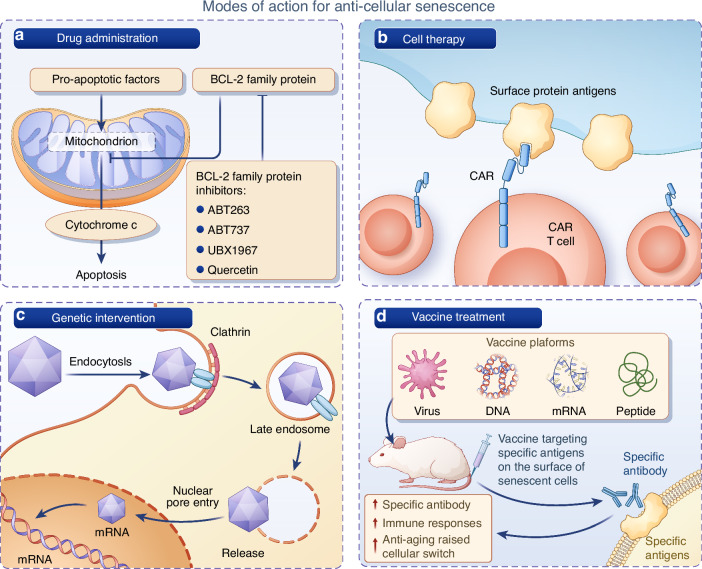
Modes of action for anti-cellular senescence. **a** Selective elimination of senescent cells by inhibiting the increase in BCL-2 and promoting senescent cell apoptosis. **b** CAR-T cells were cultured in vitro to target senescent cell-specific antigens to effectively eliminate senescent cells in vivo. **c** Genetic intervention rejuvenates senescent cells. **d** Vaccine treatment to prevent aging increased cellular senescence

The commonalities and differences in cellular senescence and other age-related mechanisms are important in understanding the role of cells in bone disease and in developing targeted therapeutic strategies. Cells in different skeletal diseases exhibit common features related to aging, such as the secretion of SASP and oxidative stress. However, there are significant differences in cellular senescence in different diseases, mainly in the specific changes of cell functions and different mechanisms of disease progression. For example, the senescence of MSCs manifests as a decrease in osteogenic differentiation capacity in OA, leading to bone loss. A decrease in the number of MSCs in OA weakens the cartilage repair capacity. However, a decrease in osteogenic capacity may reduce the ossification and alleviate the progression of OA. Senescent OBs lead to reduced bone formation, and their promotion of OCs activity in OP exacerbates bone loss, while in OA, it accelerates cartilage degeneration and inflammation. OCs enhance bone resorption, leading to OP. While in OA, their senescence contributes to the osteogenic differentiation of MSCs, exacerbating joint damage. Moreover, OCs induce H-type angiogenesis, which contributes to the alleviation of bone loss in OP, but elevated oxygen levels exacerbate the process of chondrocyte senescence in OA. In IVDD, OCs exacerbate pain through innervation. Senescent synoviocytes have been shown to lead to synovial fluid reduction and inflammation, which is manifested by synovial fibroblast dysfunction in OA, accelerating cartilage degeneration, whereas in RA, it is manifested by synovial MACs infiltration, promoting chronic inflammation and joint destruction. Currently, four principal strategies have been identified for targeting age-related cellular senescence, including drug administration, immune clearance, genetic intervention, and vaccine treatment. The translation and application of these strategies indicate directions for anti-cellular senescence in the future. Potential therapeutics for disorders that target age-related cellular senescence are shown in Fig. 5 and Table 5.

**Table 5:** Potential therapeutics for bone disorders via the targeting of senescent cells

Strategies	Agent	Species	Target of action	Outcome	References
Drug administration	D + Q	Mouse	BCL-2, BCL-X_L_	Removed senescent cells, increased longevity, and enhanced osteogenic differentiation	^219^
	ABT263	Mouse	BCL-2, BCL-X_L_	Removed senescent cells and increased longevity	^220^
	F	Mouse, human	SIRT1, IL-1β	Removed senescent cells and increased longevity	^221^
	Cardiac glycosides	Mouse	Na^+^/K^+^ ATPase pump	Removed senescent cells, increased longevity, and reduced pH	^222^
	TNF-α inhibitors	Mouse	TNF-α	Rejuvenated senescent cells and increased longevity	^224^
	IL-1 receptor antagonists	Mouse	IL-1 receptor	Rejuvenated senescent cells and increased longevity	^225^
	D + Q + F	Human	Ongoing (Phase II)		NCT04313634
	F	Human	Ongoing (Phase II)		NCT04476953
	D + Q	Human	Ongoing (Phase II)		NCT02848131
	D + Q	Human	Ongoing (Phase I/II)		NCT04785300
Cell therapy	CAR cells	Mouse	uPAR	Removed senescent cells	^229^
	Neural stem cells	Mouse	Amyloid deposition	Differentiate into effector cells	^232^
	Muscle stem cells	Mouse	Senescent muscle	Differentiate into effector cells	^233,234^
Genetic intervention	AAV	Mouse	YAP and CBX4	Rejuvenated senescent cells and increased longevity	^235,236^
	Cre-loxp Recombinase System	Mouse	KDM4B	Rejuvenated senescent cells and increased longevity	^18^
	Lentivirus	Mouse	miR-204, miR-290 and miR-302	Rejuvenated senescent cells and increased longevity	^237,238^
Vaccine treatment	VLPs vaccine	Mouse	NGF	Attenuated chronic pain and removed senescent cells	^240^
	VLPs vaccine	Human	Ongoing (Phase I)		NCT00924105
	AADvac1	Human	Ongoing (Phase II)		NCT02579252
	ACI-35	Human	Ongoing (Phase II)		NCT04445831

D dasatinib, Q quercetin, F fisetin

### Drug administration

Targeted killing of senescent cells through drugs has been shown to be effective.^219^ Researchers have observed a significant reduction in the proportion of senescent cells by simultaneously using dasatinib (D) and quercetin (Q). Aged mice treated with D and Q presented prolonged lifespan, enhanced osteogenic differentiation, and reduced aging-related bone senescence.^219^ The survival of senescent cells is closely related to the antiapoptotic proteins BCL-2 and BCL-X_L_. ABT263, an excellent specific inhibitor of BCL-2 and BCL-X, rapidly reduces the number of senescent cells when it is administered to aged mice, in which the levels of aging markers in bone cells are also significantly reduced.^220^ Fisetin (F) and cardiac glycosides are also used to reduce the number of senescent cells. F clears senescent cells from tissues, restores tissue homeostasis, and reduces aging-related pathologies.^221^ Cardiac glycosides cause extensive death of senescent cells by creating a low-pH microenvironment.^222^ In addition to directly killing cells, inhibiting SASP secretion from senescent cells is also an effective strategy for managing bone aging.^101,223^ In an aged mouse model, SASP components were successfully blocked by TNF-α inhibitors and an IL-1 receptor antagonist, which rescued bone aging.^224,225^ Drugs that target cellular senescence have shown promising anti-aging effects in animal models. However, more compelling is the generation of good data from anti-aging clinical trials. Physical function is being monitored in patients with age-related bone, lung, kidney and brain diseases after treatment with D + Q, F, or D + Q + F (NCT04313634, NCT04476953, NCT02848131, and NCT04785300).^9,226^ At least 20 clinical trials targeting age-related cellular changes are underway or planned.^227^

### Cell therapy

The off-target effects of drugs against aging-related cellular senescence lead to reduced efficacy.^228^ Cell therapy, including immune clearance and stem cell transplantation, provides better results in slowing the aging process. Immune clearance involves the removal of senescent cells by targeting specific antigens on the surface membrane of senescent cells, including the urokinase-type plasminogen activator receptor (uPAR), DPP4, and CD9 receptors.^227^ In vitro culture of uPAR-specific chimeric antigen receptor (CAR) T cells has been shown to be effective in reducing the number of senescent cells in vivo.^229^ Natural killer (NK) cells have been shown to target senescent cells through perforin-mediated granule cytotoxicity.^230^ MACs eliminate senescent cells via direct contact with senescent cells during limb regeneration in salamanders, although the exact mechanism is not fully understood.^231^ CAR-T cells, CAR-NK cells, or CAR-MACs may lead to successful removal of senescent cells with enhanced immune cell-specific targeting. The strong differentiation capacity of stem cells offers tantalizing possibilities for anti-cellular senescence. Reduced amyloid deposition and improved cognitive performance have been observed in Alzheimer’s disease mice after transplantation of neural stem cells from young mice.^232^ Transplantation of muscle stem cells has been applied in trials of myotonic dystrophy and traumatic muscle injury.^233,234^ Cell therapy promises to yield unexpected results in future anti-aging research.

### Genetic intervention

Genetic intervention rejuvenates senescent cells, restoring their youthful capacity for repair. A study revealed that the lack of YAP and chromobox (CBX) 4 in mice led to significant bone aging. After the expression of YAP and CBX4 was upregulated through adeno-associated virus (AAV) gene therapy, the mice showed with reduced signs of bone aging.^235,236^ Severe OP was observed after lysine-specific demethylase 4B (KDM4B) was knocked out in mouse BMSCs, but overexpressing KDM4B in BMSCs may have tremendous potential in OP treatment.^18^ Non-coding RNAs are closely involved in the aging process. miR-204 reduces the secretion of proteoglycans, accelerating the progression of OA.^237^ Local injection of lentiviruses containing anti-miR-204 sequences may effectively rescue OA. In addition, the overexpression of miR-290 and miR-302 reverses aging by inhibiting p21.^238^

### Vaccine treatment

Recently developed vaccines against senescent cells offer promising strategies for reversing age-related cellular senescence by targeting specific antigens and inducing immune responses.^239^ Senescence is associated with the accumulation of a number of molecular markers in senescent tissues that are absent or under-expressed in healthy tissues. Studies have revealed significantly increased levels of NGF in OA, IL-1β in type 2 diabetes mellitus (T2D), and TAU in AD, making it a potential target for anti-aging vaccines.^240–243^ In one study, recombinant NGF covalently linked to cucumber mosaic virus-derived tetanus toxoid epitopes on mosaic virus-derived virus-like particles (VLPs) significantly reversed OA-associated pain after inoculation into mice.^240^ A vaccine (NCT00924105) containing full-length recombinant IL-1β-coupled VLPs is currently being observed for its efficacy in patients with T2D, as demonstrated by ClinicalTrials.gov.^241^ Two TAU-targeted AD vaccines, AADvac1 (NCT02579252) and ACI-35 (NCT04445831), have been identified in currently conducted clinical trials, and they demonstrate unexpected translational promise.^242,243^ Despite the encouraging results observed in the current studies, further validation, including robust human clinical trials for safety, efficacy, and long-term outcomes, is needed to ascertain the anti-aging potential of these vaccines.

## Conclusion and perspectives

Cellular senescence and other age-related mechanisms has been shown to be strongly associated with the onset and progression of bone disease. The present study suggests that aging-induced cellular senescence involves collective phenotypes composed of a complex network of effector programs. Crosstalk between senescent bone cells involves changes in intrinsic senescence mechanisms, local factors, and signaling pathways. A full understanding of the commonalities and differences exhibited by senescent cells in skeletal diseases provides significant clues for potential interventions for age-related cellular senescence. The targeting of senescent changes in these cells to rescue skeletal disease has been attempted, but many key issues must be addressed before the clinical translation of these therapies can be realized. For example, while the SASP is recognized as a key driver of bone aging, the exact mechanisms by which it accelerates skeletal degeneration are not fully understood. Further investigation of the intricate crosstalk between various bone cells induced by aging is needed to reveal its impact on skeletal pathology. The identification of appropriate biomarkers is crucial for distinguishing between normal, physiologically senescent, and pathologically senescent cells, which is essential for the effective treatment of aging-related diseases.

To facilitate advancements in the field, a more comprehensive comparative analysis of aging-related cellular senescence in different skeletal diseases is needed to identify both common mechanisms and those changes that are specific to individual diseases. This will provide a theoretical basis for the design of universal and customized treatments. From a clinical perspective, current therapeutic strategies, including drug administration, cell therapy, genetic intervention, and vaccine treatment, require further refinement. Future studies should integrate multiple therapeutic modalities to comprehensively regulate intercellular and intracellular crosstalk networks, with an emphasis on improving the stability, efficacy and safety of interventions. In addition, given the heterogeneity of the individual aging process, a precision medicine approach should be prioritized to tailor anti-aging therapies to specific molecular and cellular aging profiles. By addressing these challenges, future research could bridge the gap between basic research and clinical applications, ultimately providing more effective and personalized interventions for aging-related diseases.

Cellular senescence and other age-related mechanisms in skeletal diseases not only contribute to the development of a variety of skeletal diseases, but also tend to affect organ dysfunction throughout the body, resulting in a deleterious cascade effect. This review also provides important insights into the field of systemic age-related diseases. Understanding the mechanisms associated with cellular senescence in bone tissue may provide new perspectives for treating age-related dysfunction in other organs. In addition, therapeutic strategies targeting senescent cells have great potential for improving the quality of life of elderly individuals and thereby promoting healthy aging. From a societal perspective, age-related diseases generate significant healthcare costs and long-term care needs. Effective interventions to delay the onset of age-related diseases can reduce hospitalization rates, decrease the need for surgical interventions, reduce the reliance on assisted care, and ultimately benefit both the individual and the healthcare system. Future research that integrates skeletal aging with systemic aging diseases may pave the way for comprehensive anti-aging therapies that improve longevity and overall health.
